# Efficacy of supplementary vitamin D on improvement of glycemic parameters in patients with type 2 diabetes mellitus; a randomized double blind clinical trial

**DOI:** 10.12861/jrip.2014.10

**Published:** 2013-11-30

**Authors:** Hamid Nasri, Saeed Behradmanesh, Ahmad Reza Maghsoudi, Ali Ahmadi, Parto Nasri, Mahmoud Rafieian-Kopaei

**Affiliations:** ^1^Department of Nephrology, Division of Nephropathology, Isfahan University of Medical Sciences, Isfahan, Iran; ^2^Department of Internal Medicine, Shahrekord University of Medical Sciences, Shahrekord, Iran; ^3^Department of Epidemiology, Shahid Beheshti University of Medical Sciences, Tehran, Iran; ^4^Faculty of Medicine, Isfahan University of Medical Sciences, Isfahan, Iran; ^5^Medical Plants Research Center, Shahrekord University of Medical Sciences, Shahrekord, Iran

**Keywords:** Type 2 diabetes patients, Vitamin D, HbA1c

## Abstract

**Introduction: ** Studies have revealed the association between vitamin D deficiency and changes in blood glucose and insulin levels as well as sensitivity of the target tissues to insulin.

**Objective:** In this study, we examined the effect of adding vitamin D (cholecalciferol ; 50,000 units) to therapeutic regimen of T2DM patients compared to placebo on regulating the blood glucose and glycemic parameters.

** Patients and Methods:** This study was a double blind clinical trial conducted on 60 type 2 diabetes mellitus (T2DM) patients. Exclusion criteria were taking calcium, vitamin D supplements or any drugs effecting calcium and vitamin D metabolism in the past 6 months. Serum 25-Hydroxy vitamin D [25(OH)D] level was measured with ELISA method. Patients were administered weekly vitamin D supplementation (50000 units) for 12 weeks.

**Results:** There was no significant relation between HbA1c and 25(OH)D level prior the study (p> 0.05). After intervention, 25(OH)D level in interventional group was significantly higher compared to that of control group. HbA1c in male interventional group was significantly less than that of control group (p= 0.0068).

**Conclusion:** Weekly vitamin D supplementation had beneficial effect on glycemic parameters in male type 2 diabetic patients.

**Implication for health policy/practice/research/medical education:**

In this study we found that weekly vitamin D supplementation (cholecalciferol; 50,000 units for 12 weeks) had beneficial effect on HbA1c of male type 2 diabetic patients. Thus, oral vitamin D may help in improvement of glycemic control in these patients.

## Introduction

Diabetes mellitus is one of the most common metabolic diseases in the whole world. Over 150 million people are diagnosed with this disease worldwide and it is predicted that this number will reach to over 300 million people by 2025 (1). Vitamin D is a fat soluble vitamin which was discovered and named calciferol in 1930. In fact this vitamin is a hormonal precursor which its end product is built up inside the body (1,2). Apart from the crucial role of this vitamin in bones, this vitamin plays an essential role in autoimmune disorders as well as preventing colorectal, breast and prostatic cancer through preventing cell proliferation (3). Although 1,25(OH)2 vitamin D3 is important in calcium homeostasis, several investigations have revealed the effect of this vitamin on function of the immune system and also type 1 and type 2 diabetes mellitus (T2DM) (4). Studies have revealed the association between vitamin D deficiency and changes in blood glucose and insulin levels as well as sensitivity of the target tissues to insulin (5,6). This vitamin has many functions in the body and its receptors are present in over 30 different tissues such as pancreas, heart and lymphocytes (4-6). Vitamin D might help controlling diabetes mellitus through increasing insulin secretions and increasing sensitivity to this hormone (4-6). Several researches have evaluated the association between vitamin D deficiency and glucose intolerance prevalence of T2DM. Various studies have shown a negative association between serum vitamin D level and glucose intolerance, while some others have not found any correlation (7). Only a few studies have assessed the effect of vitamin D on changes in blood glucose levels and insulin sensitivity, reporting controversial results (7-10). Many studies have demonstrated positive effect of vitamin D supplements on controlling hyperglycemia in diabetic patients. However, these studies are mainly conducted on animals (8-11). Studies on rats have revealed the direct effect of vitamin D on insulin secretion (6-12). Yet, the results of precisely designed randomized double blind controlled trials are not consistent with this finding (10-13).

Various studies have demonstrated the presence of 1-α hydroxylase and vitamin D receptors (VDR) on beta cells of pancreas. This enzyme converts 25(OH)D to 1, 25(OH)2 D3 (7-13). These findings suggest the possible effect of vitamin D on glucose homeostasis (10-16). Therefore, if the beneficial effect of vitamin D therapy on controlling diabetes is approved, it could be used as an adjunct therapy in treating diabetes. Furthermore, it is revealed that vitamin D deficiency, even in developed countries is more than what predicted (12-17).

## Objectives

In this study, we examined the effect of adding vitamin D to therapeutic regimen of T2DM patients compared to placebo on regulating the blood glucose and glycemic parameters.

## Method and Materials

### Patients

This study was a randomized, double-blind, placebo controlled clinical trial on 60 patients diagnosed with T2DM referred to endocrinology clinic of Shahrekord University of Medical Sciences in 2011. The patients by computer-generated randomly permutated codes (prepared by WHO/Geneva) were allocated into two equal groups of 30. The inclusion criteria were definite diagnosis of T2DM according to standard criteria, no renal or hepatic disease or any other chronic illness based on history and physical examinations. Exclusion criteria were taking calcium, vitamin D supplements or any drugs effecting calcium and vitamin D metabolism in the past 6 months.

### Laboratory tests

First, the blood level of 25-Hydroxy vitamin D [25(OH)D] was measured in all the patients. One group received oral vitamin D (cholecalciferol), 50000 units per week for 12 weeks, while the other group received placebo for the same period of time. In all patients, fasting blood sugar (FBS), blood sugar (BS), 2 hour postprandial blood sugar (2-hpp) and HbA1c were measured before and after drug therapy. Serum 25(OH)D was measured with ELISA method with sensitivity of 5 nol/L (2 ng/ml) and specificity of 100 percent by Stat fax 2100 produced by Awareness Company (The United States). Serum 25(OH)D of below 50 nmol/L was considered deficient, between 50 and 70 nmol/L insufficient, between 75 and 250 nmol/L sufficient and over 250 nmol/L potential intoxication. HbA1c was measured using column chromatography method with Nyco card reader II made in Norway. FBS, 2-hpp, BS were measured using spectrophotometer by Erba-XL 300 made in Germany.

### Ethical issues

The research followed the tenets of the Declaration of Helsinki. Written informed consent was obtained from all patients. This study was approved by Ethical Committee of Shahrekord University of Medical Science. This study was registered in Iranian Registry of Clinical Trials (IRCT) and achieved the code of IRCT201011185191N6, too.

### Statistical analysis

The data was analyzed with Stata software version 12 (Stata Corp, College Station, Tex) using student t-test, paired t-test, Pearson correlation and Chi square tests. P values of less than 0.05 was assumed to be significant (p<0.05).

## Results

A total number of 60 type 2 diabetic patients were randomly assigned into two groups of intervention and control, each group consisting of 30 patients. The intervention group received vitamin D and the control group received placebo. The age of the patients ranged from 34 to 76 with the mean (SD) age of 55 (10.7) years. There was not significant difference of age between groups (p= 0.88). Seventeen patients (28.3%) were male. The female to male ratio was not statistically significant (p= 0.58). Five patients had vitamin D deficiency (8.3%), 27 (45%) had insufficient levels of vitamin D and in 28 (45%) patients vitamin D levels were within normal limits. None of the patients had vitamin D intoxication. There was no significant relation between HbA1c and 25(OH)D level prior the study (p=0.379). Table 1  demonstrates the laboratory parameters of both groups before and after the intervention. After intervention, 25 (OH)D level in interventional group was significantly higher compared to that of control group. HbA1c serum value of male diabetic patients in interventional group was less than that of control group (p=0.0068).

**Table 1:** Mean (SD) of HbA1C and 25(OH)D and other laboratory parameters in relation to sex

**Time of measurement** **Group**	**Intervention Group**			**Control Group**			Comparison Between Group P value*	
	before Mean (SD)	after Mean (SD)	P value	before Mean (SD)	after Mean (SD)	P value	Interventional	Control
HbA1c	Male	7.67 (0.5)	6.78 (0.9)	0.0068*	7.41 (0.4)	6.97 (0.7)	0.22	0.019*	0.214
	Female	7.65 (0.4)	7.45 (1.3)	0.52	7.68 (0.5)	7.67 (1)	0.95	0.53	0.959
	Total	7.65 (0.4)	7.62 (0.52)	0.799	7.22 (1.2)	7.51 (1)	0.349	0.071	0.551
25(OH)D (nmol/L)	Male	82.5 (49)	167.8 (32)	0.001*	93.3 (67)	94.5 (55)	0.83	0.002*	0.971
	Female	84.6 (54)	162.2 (67)	0.001*	109.5 (64)	122.2 (103)	0.4	0.003*	0.619
	Total	83.9 (52)	164 (57)	0.001*	105.7 (64)	115.8 (94)	0.39	0.001*	0.632

*P <0.05 comparing the data of each group before and after intervention

## Discussion

In this study, we found that serum HbA1c value was significantly less than that of control group in male interventional group. It means that, vitamin D therapy has had beneficial effect on the control of blood sugar in male diabetic patients. Vitamin D has evolved widespread interest in the pathogenesis and prevention of diabetes. Various prospective cohort investigations have shown that serum vitamin D level is inversely related to risk of T2DM (18).****Sadek *et al*. conducted a study to evaluate the potential therapeutic efficacy of Vitamin D in preventing the detrimental effects of both types of diabetes mellitus, on 50 male Wistar rats. They found that, Vitamin D improved the deleterious biochemical impact of diabetes mellitus, likely by increasing insulin secretion and sensitivity, improving the β-cell function, and decreasing the number of pro-inflammatory cytokines and insulin resistance (19). Altered expression of glucose transporters is a major characteristic of diabetes (12-16). Tamilselvan *et al.,* aimed to investigate the effect of vitamin D in the overall regulation of muscle cell glucose transporter expression. Treatment of myoblasts with 10-7 M calcitriol for 24 h showed a significant increase in glucose transporter type 1 (GLUT1), GLUT4, vitamin D receptor (VDR), and insulin receptor expression in type 1 and 2 diabetic model compared to control group. The results showed a potential anti-diabetic function of vitamin D on GLUT4, VDR, GLUT1 and insulin receptor by improving receptor gene expression suggesting a role for vitamin D in regulation of expression of the glucose transporters in muscle cells (20). To assess the relationship between 25 (OH) vitamin D level and microvascular complications in patients with T2DM, 136 patients were enrolled in the cross-sectional study of Ahmadieh *et al.* They found, serum 25 (OH) vitamin D correlated negatively with HbA1c. They concluded that, low 25 (OH) vitamin D level was an independent predictor of HbA1c, diabetic neuropathy, and diabetic retinopathy in patients with T2DM (21). However, Tai *et al.* found that in short term, correcting of vitamin D level had no significant impact on blood glucose level, plasma insulin, insulin sensitivity or oral glucose tolerance test in vitamin D deficient patients who had not diabetes (10). Iyengar *et al.* measured fasting blood glucose level, C peptide and insulin as well as 2 hours blood sugar after 75 gram oral glucose intake in a Hispanic-American and Angolan population in order to assess the role of vitamin D binding globulin on regulating blood glucose level. The study showed a significant positive correlation between fasting blood sugar and vitamin D binding peptide genotype (22). Hossein-Nezhad *et al.* evaluated 741 pregnant women in a cross-sectional survey. They found, a significant association between insulin resistance index and insulin sensitivity index with serum vitamin D level (after adjusting for BMI) (23). Additionally, active form of vitamin D is a protective factor against type 1 diabetic none-obese rats, which was found in the study of Giulietti *et al*. They also showed that incidence of type1 DM was higher in none-obese rats which were exposed to vitamin D deficiency in their early life (24). Likewise, Champe *et al.* found a negative association between serum vitamin D levels and fasting blood glucose and postprandial glucose levels too (25). As seen above, most researches indicating the impact of serum vitamin D level and blood glucose and insulin resistance are either experimental or cross-sectional epidemiologic studies or on non-diabetic patients.

## Conclusion

In our study, on 60 type 2 diabetes patients, who were randomly assigned into two equal groups of intervention and control, we found that weekly vitamin D supplementation (50000 units for 12 weeks) had beneficial effect on HbA1c of male type 2 diabetes mellitus patients. However studies with larger sample size and longer duration of intervention or higher dosage of vitamin D supplements suggests.

## Authors’ contributions

Main draft write up and editing by SB, PN, HN and ARM. Important intellectual content and critical revision by MRK and AA.

## Conflict of interests

The author declared no competing interests.

## Ethical consideration

Ethical issues (including plagiarism, misconduct, data fabrication, informed consent, double publication) have been completely observed by the authors.

## Funding/Support

This paper has been derived from the residential thesis. This study was granted by Shahrekord University of Medical Sciences (Grant# 844).
